# DNA Barcodes and Morphology Reveal Two New Species of the Genus *Prochas* Walkley, 1959 (Ichneumonidae, Campopleginae), from China †

**DOI:** 10.3390/insects15120968

**Published:** 2024-12-04

**Authors:** Yuanyuan Han, Kees van Achterberg, Xuexin Chen

**Affiliations:** 1 State Key Lab of Rice Biology, Zhejiang University, Hangzhou 310058, China; yyhan6@zju.edu.cn (Y.H.); kees@vanachterberg.org (K.v.A.); 2 College of Biology and Food Engineering, Chuzhou University, Chuzhou 239000, China; 3 Zhejiang Provincial Key Laboratory of Biology of Crop Pathogens and Insects, Zhejiang University, Hangzhou 310058, China; 4 Institute of Insect Sciences, College of Agriculture and Biotechnology, Zhejiang University, Hangzhou 310058, China; 5 Ministry of Agriculture Key Lab of Molecular Biology of Crop Pathogens and Insects, Zhejiang University, Hangzhou 310058, China

**Keywords:** DNA barcoding, integrative taxonomy, oriental and eastern palearctic, parasitoids

## Abstract

DNA barcoding is an effective modern tool in taxonomy, evolutionary biology, and biodiversity research. Many new species have been discovered and described with DNA barcodes as part of their diagnostic features. We combined morphological examination and molecular species delimitation of the mitochondrial cytochrome c oxidase 1 (*COI*) gene using the automatic barcode gap discovery (ABGD) to investigate species boundaries. The genus *Prochas* Walkley (Hymenoptera, Ichneumonidae, Campopleginae) was first reported from China and is new for the Oriental and Eastern Palearctic regions. Using an integrative taxonomy method, two new species *P. rugipunctata* sp. nov. and *P. striata* sp. nov. are hereby described and illustrated. A key to the world species and a distribution map are provided.

## Simple Summary

Species of Campopleginae develop as koinobiont endoparasitoids, the vast majority of which are solitary parasitoids of Lepidoptera, which have proven to be effective in the biological control of pests. The study of this subfamily is minimal, considering its importance and distribution. The poorly studied genus *Prochas* includes three species previously reported from the neotropical region, and there is little information available about this species distribution and biology. For the study of this scarce genus, we acquired specimens of this genus from the Parasitoid Hymenoptera Collection of the Institute of Insect Sciences, Zhejiang University. Based on morphology and DNA barcodes, *Prochas rugipunctata* sp. nov. and *Prochas striata* sp. nov. were described and illustrated. In addition, this study provided a morphological diagnosis for *Prochas* as well as a key to world species of this genus. Together with the DNA barcodes presented here, this study provides essential and useful information for the species identification of *Prochas*.

## 1. Introduction

Over the past decade, the use of short standardized genetic markers for species identification, so-called DNA barcoding [1,2], has proven effective in biodiversity assessments and taxonomic revisions [3,4,5,6,7,8,9]. DNA barcodes have also been used efficiently to detect hidden species diversity in Ichneumonidae [10].

Campopleginae are a large subfamily with more than 2200 accepted species names worldwide, containing several species important for the biological control of insects [11,12,13,14]. For example, *Diadegma semiclausum* parasitizes the diamond back moth, *Plutella xylostella* [15], and *Venturia canescens* is important in the control of stored product pests, notably *Ephestia kuehniella* and *Plodia interpunctella* [16,17,18,19]. However, this is a relatively poorly studied group, a lot of species remain to be described, and it is likely the second largest subfamily after Phygadeuontinae [20].

The genus *Prochas* Walkley (in Short), 1959 (Ichneumonidae, Campopleginae), is a small genus with three valid species, which had previously only been reported in the neotropical region [21,22,23,24,25]. The biological information is scarce, and only the Caribbean type species *P. theclae* has been reared from the butterfly *Ostrinotes empusa* (Hewitson, 1867) (Lycaenidae) on cacao (Walkley (in Short) 1959).

The two new species *Prochas rugipunctata* sp. nov. and *Prochas striata* sp. nov. match the generic diagnosis well and are characterized by having their body covered with dense silvery setae, the inner margin of the eye being slightly indented opposite to the antennal socket, the propodeum having a superomedial areolate area, the fore wing not having an areolet, the hind basitarsus not having a median ventral row of setae, the occipital carina joining the hypostomal carina at the mandible base, and the clypeus having a transverse-carina medially, but being abruptly declivous apically, and having an apical margin that is truncate and reflexed [21,24,26,27].

**Figure 1:**
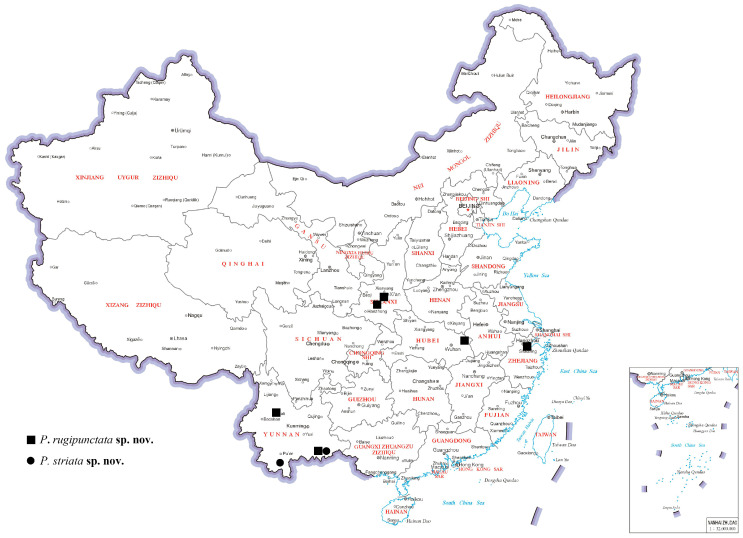
Distribution map of the new species from China (map of China from http://bzdt.ch.mnr.gov.cn/, format: GS(2019), number1686).

We describe and illustrate two new species by combining morphology and DNA barcodes, and we provide a key to the world species. A distribution map is included below (Figure 1).

## 2. Materials and Methods

### 2.1. Origin of the Specimens

This study is based on specimens preserved in the Parasitoid Hymenoptera Collection of the Institute of Insect Sciences, Zhejiang University (ZJUH), which contains about 0.6 million pinned specimens and about the same number of specimens in alcohol, collected from all over China.

### 2.2. Morphological Study

The terminology and measurements used follow Broad et al. [20]. All descriptions and measurements were determined under a ZEISS Stemi 305 microscope, and all the figures were made using a digital KEYENCE VHX-7000C microscope (KEYENCE, Osaka, Japan). The type specimens were deposited in the Parasitic Hymenoptera Collection of Zhejiang University (Hangzhou, China, ZJUH).

### 2.3. DNA Extraction, Amplification, and Sequencing

Nine specimens of two Chinese *Prochas* species were sequenced in this study, and seventeen sequences of the genus *Prochas* were downloaded directly from Bold (Barcode of Life Data System (https://boldsystems.org/, accessed on 21 October 2024). In addition, we downloaded BIN data of *Cryptophion inaequalipes* as the outgroup (Table 1). Specimens for DNA extraction were kept in absolute ethyl alcohol at 4 °C and protected from light before being studied. Genomic DNA was extracted using Qiagen DNA Blood and Tissue Kits, following the standard protocol, except for the fact that the final elution volume in this study was 60 μL. Each individual sample was obtained by whole-body soaking, without destroying the samples, recovering the specimens after extraction. The universal primers LCO1490 and HCO2198 were used to amplify the standard 658 bp mitochondrial COI barcode region [28]. Polymerase chain reaction (PCR) amplifications were performed in a 20 μL volume including 10 μL of KOD mix, 1μL of forward primer, 1ul of reverse primer, 3 μL of template DNA, and 5 μL of deionized H_2_O. The PCR product was purified and sequenced in both directions at the Zhejiang Sunya Biotechnology Co., Ltd., Hangzhou, China.

### 2.4. Data Analysis

Mitochondrial COI sequences were aligned by codons using MUSCLE implemented in MEGA 11 [29,30]. A maximum-likelihood (ML) tree was constructed based on nine obtained *COI* genes and seventeen *Prochas* COI genes downloaded from BOLD, with species *Cryptophion inaequalipes* as an outgroup. Using PartitionFinder ver 2.1.1 [31], the best fitted model was identified, and then cluster analysis was carried out through the maximum-likelihood (ML) method using MEGA 11. Bootstrap analysis was performed based on 1000 resampling. The ML tree was visualized using iTOL v.5 [32]. In addition, genetic distances for each *COI* sequence were calculated using the Kimura 2-parameter (K2P) model in MEGA 11. Pairwise similarity scores were determined with SDTv1.3 (Sequence Demarcation Tool Version 1.3) [33], which was also used to generate a color-coded matrix displaying these scores.

The Automatic Barcode Gap Discovery (ABGD) analysis was employed for species delimitation. The ABGD analysis was conducted via a web interface (https://bioinfo.mnhn.fr/abi/public/abgd/abgdweb.html, accessed on 28 September 2024), using the K2P model to classify species based on genetic distances. The value of the relative gap width (X) was set as 1.5, and prior intraspecific divergence (*p*) was set as 0.001–0.1 [34].

## 3. Results

### 3.1. Key to Species of Prochas Walkley (Females)

1. This species presents an entirely black metasoma, a punctate metapleuron and a strong and complete juxtacoxal carina, a propodeum with a trapezoidal superomedial area, a straight ovipositor which is 0.6× the length of the hind tibia, and a fore wing with a 1cu-a vein opposite the M&RS vein. It is found in Trinidad and Tobago.....................................................................................................................................................................................................................................................................................................................................*P. theclae* Walkley, 1959

-. The metasoma is largely orange or reddish brown. The metapleuron is usually not punctate and the juxtacoxal carina is variable. Their propodeum has non-trapezoidal superomedial area. This species ovipositor is straight or upcurved, more than 0.6× the length of the hind tibia. Their fore wing has a 1cu-a vein, usually distal to the M&RS vein............................................................................................................................................................................................................................................................................................................2

2. This species’ head is mostly granulose and its metapleuron presents a developed juxtacoxal carina. The scape and pedicel are ventrally light yellow, and the mandible, tegula, and clypeus are apically yellow............................................................................................................................................................................................................................................................................................................3

-. The head is mostly rugose and punctate, their metapleuron does not present a juxtacoxal carina, and their scape, pedicel, mandible, tegula, and clypeus are black (in *P. rugipunctata*, the mandible is medially yellowish brown)............................................................................................................................................................................................................................................................................................................4

3. This species’ antenna has 26 flagellomeres, with the first flagellomere being 3.0× as long as it is wide. The lateral ocelli are separated from the eye by a distance that is 0.6× their diameter, and the distance between lateral ocelli is 2.5× their diameter. The lower half of the mesopleuron is punctate, while the propodeum has a coffin-shaped superomedial area. The fore coxa does not have a ventral carina, and the fore wing has a vein 1cu-a distal to vein RS&M. The ovipositor is slightly upcurved and as long as the hind tibia. This species can be found in Brazil..........................................................................*P. brasiliensis* Onody & Penteado-Dias, 2015

-. The antenna has 31 flagellomeres, with the first flagellomere 5.0× as long as it is wide. The lateral ocelli are separated from eye by a distance that is 0.4× their diameter, while the distance between lateral ocelli is 1.5× their diameter. The lower half of the mesopleuron is rugose and punctate, and the propodeum has a hexagonal superomedial area. The fore coxa has a ventral carina, and the fore wing has a vein 1cu-a opposite to vein RS&M. The ovipositor is straight and 0.65× the length of the hind tibia. This species can be found in Brazil............................................................................................................................................................................................................*P. shimborii* Onody & Penteado-Dias, 2015

**Figure 2:**
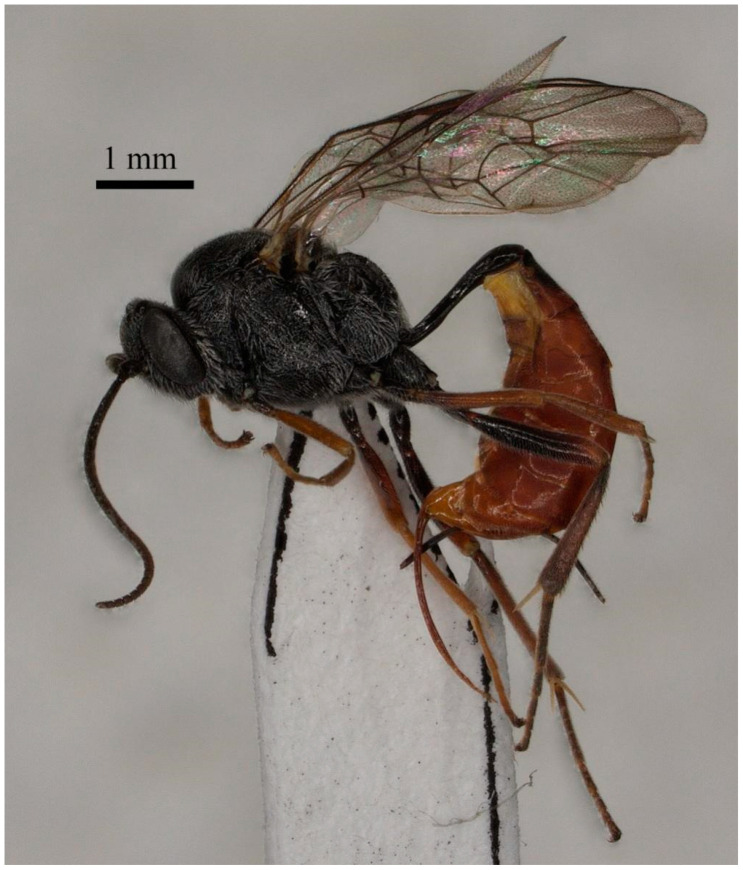
*Prochas rugipunctata* sp. nov., holotype, female, and habitus.

**Figure 3:**
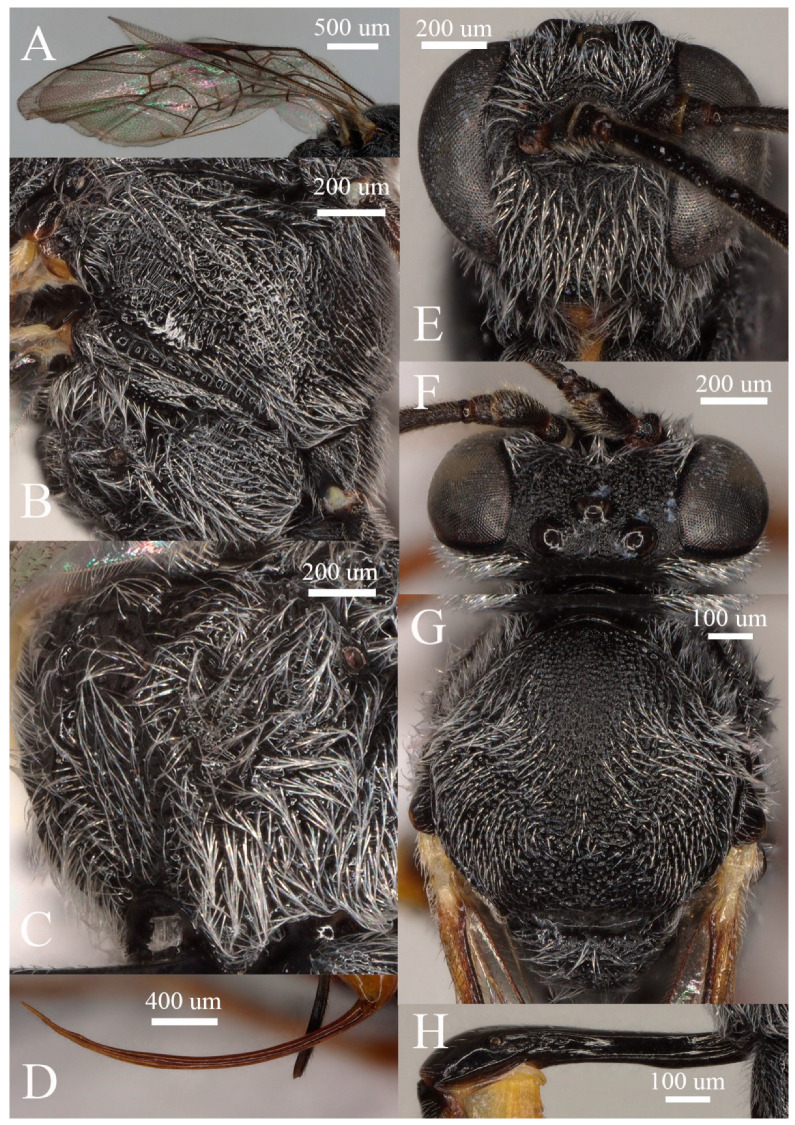
*Prochas rugipunctata* sp. nov. holotype of a female specimen: (**A**) fore wing, (**B**) mesosoma, (**C**) lateral view of the propodeum, (**D**) dorsal view of the ovipositor, (**E**) lateral view of the head, (**F**) anterior view of the head, (**G**) dorsal view of the mesoscutum, and (**H**) dorsal lateral view of the first metasomal segment.

4. This species’ clypeus is rugose and punctate, like the malar space (but the rugae here are weakly developed). The upper half of the mesopleuron is rugose and punctate, becoming rugose along the epicnemial carina (Figure 3B). The superomedial area is ca. 0.7× as long as it is wide (Figure 3C). The mandible is medially yellowish brown (Figure 3E), while the fore and middle trochanters, hind trochanter, trochantellus, and basal 0.9 of the femur are black. The hind tibia is blackish brown basally and apically, like the tarsomeres (Figure 2), while the first segment and second tergite 0.7 of the metasoma are black (Figure 2). This species can be found in China....................................................................................................................................................................................*P. rugipunctata* sp. nov.

**Figure 4:**
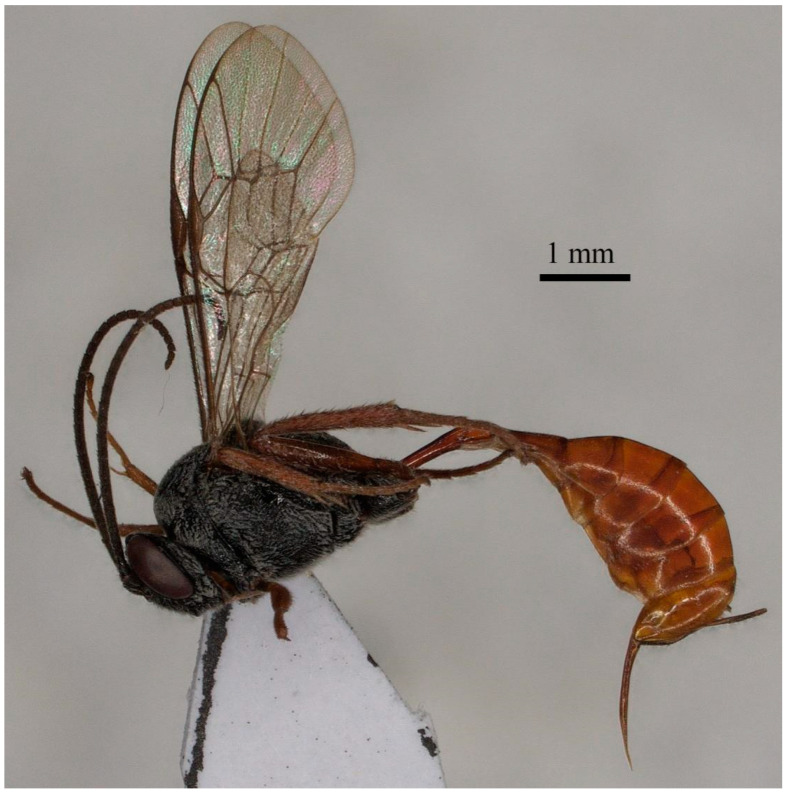
*Prochas striata* sp. nov. holotype, female, and habitus.

**Figure 5:**
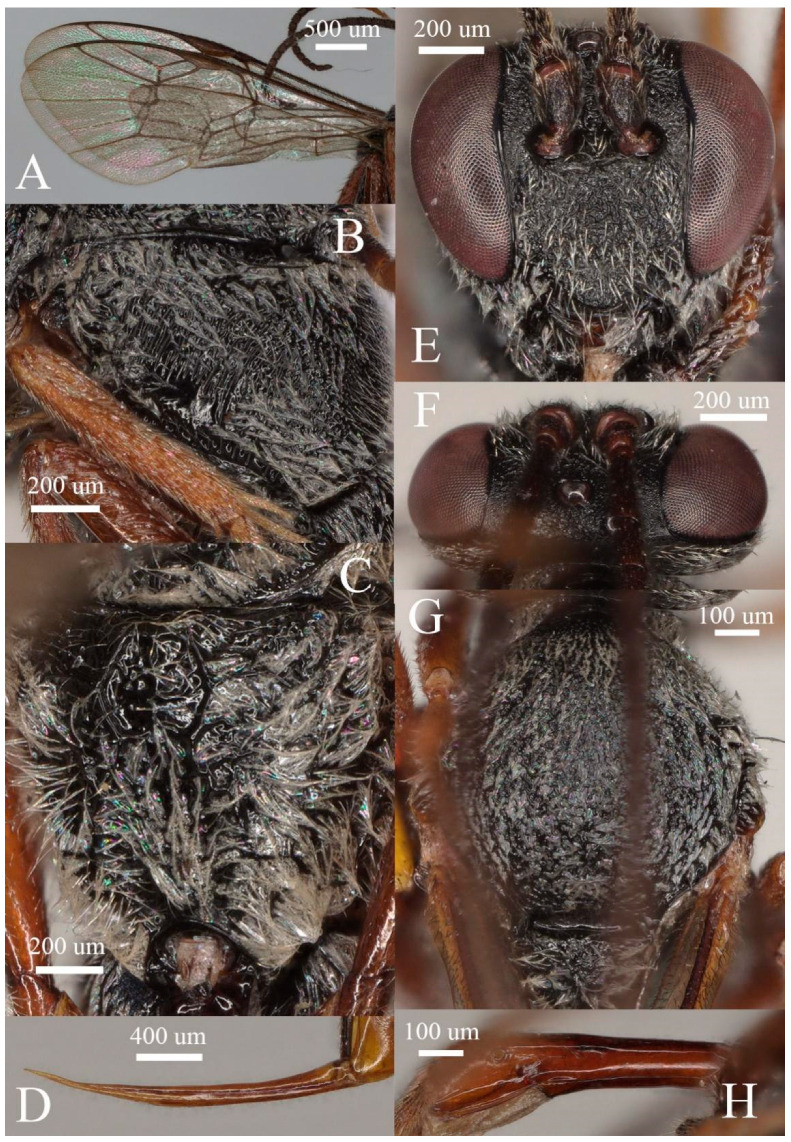
*Prochas striata* sp. nov. holotype for a female specimen: (**A**) fore wing, (**B**) mesosoma, (**C**) lateral view of the propodeum, (**D**) dorsal view of the ovipositor, (**E**) lateral view of the head, (**F**) anterior view of the head, (**G**) dorsal view of the mesoscutum, and (**H**) dorsal and lateral view of the first metasomal segment.

-. This species’ clypeus is granulose and rugulose, while the malar space is nearly smooth. The upper half of the mesopleuron, extending toward the epicnemial carina, is striate and rugose (Figure 5B). The superomedial area is ca. 0.85× as long as it is wide (Figure 5C). The mandible is black (Figure 5E), while the fore and middle trochanters and hind leg, except for the coxa, are orange brown (Figure 4). The metasoma is wholly orange brown (Figure 4). This species can be found in China.................................................................................................................................................................................... *P. striata* sp. nov.

### 3.2. Morphological Diagnosis

The genus described below is *Prochas* Walkley (in Short, 1959).

*Prochas* Walkley is described in Short (1959: 391). The type species is *Prochas theclae* Walkley (described in Short, 1959, by monotype and original designation).

This is a medium-sized species whose body is covered by dense silvery setae. The clypeus has a carina which is medially transverse, but abruptly declivous apically, and the apical margin of the clypeus is truncate and reflexed. The mandible has a strong lamella, and the upper tooth is as long as the lower tooth. The eye is slightly indented opposite to the antennal socket. The occipital carina reaches the hypostomal carina at the base of the mandible. Meanwhile, the propodeum has an areolate superomedial area, separated from area petiolaris. The hind tarsal segments do not present a row of ventral setae medially, and the fore wing does not have an areolet. Meanwhile, the hind wing has CU&cu-a that is vertically declivous and not intercepted. The first metasomal segment does not have a glymma, and the ovipositor is straight and then upcurved, without a subapical notch.

#### 3.2.1. *Prochas rugipunctata* sp. nov. (Figure 2 and Figure 3)

The following features characterize female specimens of this species. *Prochas rugipunctata* can be distinguished from known species of *Prochas* by the following combination of characters: rugose and punctate clypeus and malar space; a rugose and punctate upper half of the mesopleuron, becoming rugose along the epicnemial carina; rugose and punctate mesoscutum, metanotum, and metapleuron; an absence juxtacoxal carina; a mandible that is medially yellowish brown; black fore and middle coxa, trochanter, and trochantellus; black hind trochanter, trochantellus, and basal 0.9 of the femur; black tegula; and black first segment and second tergite 0.7 of the metasoma, while the remainder of the metasoma is orange brown.

We examined the following materials: Holotype: CHINA • ♀; Yunnan, Dali Cangshan; 5.V.2009; Man-Man Wang leg.; No. 202019698. Paratypes: CHINA • 1♀; Hubei, Dabieshan; 23.VI.2014; Zhen Liu leg.; No. 202019699 (BOLD ID: GBMNE65794-22) • 1♀; Shaanxi, Qinling Niubeiliang Natural Reserve; 30.VIII.2013; Bin-Bin Tu leg.; No. 202019700 (BOLD ID:GBMNE65792-22) • 2♀; Shaanxi, Foping; 3.VII.2013; Jiang-Li Tan leg.; No. 201304946 (BOLD ID: GBMNE65797-22), 201304906 (BOLD ID: GBMNE65798-22) • 1♀; Shaanxi, Niubeiliang Guanghuojie; Juan Mao leg.; No. 202019702 (BOLD ID: GBMNE65796-22) • 1♂; Yunnan, Maguan, 20.IX.2019; Malaise trap; No. 202019703 • 1♀; Zhejiang, Longquan Fengyangshan, 12.VI.2008; Sheng-Long Liu leg.; No. 202019701 (BOLD ID: GBMNE6Q5795-22).

The following is a description of the female (Figure 2) holotype, with a body length of 7.5 mm and a fore wing length of 5.5 mm.

The head presents an antenna with 28 flagellomeres, with the first flagellomere being ca. 2.7× as long as it is wide. The face (Figure 3E) is rugose and punctate. The clypeus (Figure 3E) has a medially transverse carina, which apically becomes abruptly declivous, rugose and punctate, and convex, while the apical margin is truncate and reflexed. The malar space is rugose and punctate with rugae which are weakly developed; its length is ca. 0.6× the basal width of the mandible. The upper tooth of the mandible is equal to the lower tooth, with a strong lamella. The frons (Figure 3E) are rugose and punctate, without a median carina. The vertex (Figure 3F) is granulose. The interocular region (Figure 3F) is rugose and punctate. The interocular distance is 2.5× the ocello-ocular distance and 2.3× the distance between the median and lateral ocelli, while the distance between the lateral ocelli and the eyes is ca. 0.6× as long as the diameter of the ocellus. The temple mat is ca. 0.5× as long as the eyes in the lateral view. The occipital carina is evenly arched, reaching the hypostomal carina at the mandible’s base.

The mesosoma presents a pronotum that is dorsally punctate and transversely striated on the side. The mesoscutum (Figure 3G) is rugose and punctate, with rugae stronger on the notaulic area. The scutellum and metanotum are rugose and punctate. The mesopleuron (Figure 3B) is rugose and punctate on the upper half, becoming rugose along the epicnemial carina, and transversely striated below the tegula, the lower half being rugose and punctate, with punctures separated from one another at a distance which is, at most, the same as their diameter. The speculum is smooth and shiny. The metapleuron (Figure 3B) is rugose and punctate, without a juxtacoxal carina. The propodeum (Figure 3C) has a short and trapezoid area basalis, while the superomedial area is hexagonal, ca. 0.7× as long as it is wide, and separated from the area petiolaris. The superomedial area is rugose, while the area petiolaris, area dentipara, area externa, and area lateralis are rugose and punctate. All carinae are strongly developed. The propodeal spiracle is oval, connected to the pleural carina by a distinct carina.

The fore wing (Figure 3A) do not have an areolet. The portion of vein M between 2rs-m and 2m-cu is ca. 0.5× as long as 2rs-m. The marginal cell short vein RS is ca. 2.0× longer than vein 2r&RS. Vein 1cu-a is slightly distal to M&RS by 0.2× of its length. The external angles of the second discal cell are acute (55°). The hind wing has a declivous CU&cu-a, and 2-CU is not connected with CU&cu-a.

Regarding the legs, the hind femur is 5.5× as long as it is wide, while the inner spur of the hind tibia is ca. 0.6× as long as the first tarsomere of the hind tarsus. The tarsal claws are pectinate.

In the metasoma, the first segment (Figure 3H) does not have a glymma and is ca. 3.8× longer than the width of the postpetiole. The petiole is ca. 3.3× longer than the postpetiole. The second tergite is ca. 0.5× as long as the first tergite and 1.5× longer than its apical width. The thyridium is oval, and its distance from the basal margin of the tergite is ca. 0.6× its length. The third tergite is as long as its apical width. The ovipositor (Figure 3D) is upcurved, ca. 1.3× the apical depth of the metasoma and ca. 1.3× the length of the hind tibia, without a notch on the upper valve. The ovipositor sheath is ca. 0.6× as long as the hind tibia.

The color of the scape and pedicel, basal 0.6 of the mandible, and tegula is black, whereas the mandible is apically brown, and the remainder of the mandible is yellowish brown. The fore coxa, trochanter, and trochantellus are black, while the remainder of the fore leg is orange brown. The mid coxa, trochanter, trochantellus, and femur are basally black, and the remainder of the mid leg is orange brown. The hind coxa, trochanter, trochantellus, and basal 0.9 of the femur are black, whereas the tibia (basally and apically) and tarsomeres are blackish brown. The remainder of the hind leg is orange brown. The first segment and second tergite 0.7 of the metasoma are black, while the remainder of the metasoma is orange brown.

Upon comparative diagnosis, this species is similar to *P. striata* sp. nov. but differs from the latter by having a rugose and punctate clypeus and malar space, the latter having weakly developed rugae. It also differs due to having a mesopleuron that is rugose and punctate on its upper half, becoming rugose along the epicnemial carina. Another difference is the superomedial area being ca. 0.7× as long as it is wide, a mandible which is medially yellowish brown, and black fore and mid trochanters, hind trochanter, trochantellus, and basal 0.9 of the femur. Moreover, its tibia, both basally and apically, and tarsomeres are blackish brown, and the first segment and second tergite 0.7 of the metasoma are black.

Regarding distribution, this species can be found in China (Hubei, Shaanxi, Yunnan, Zhejiang).

Regarding variations, the antenna can have 28–30 flagellomeres, the first flagellomere is 2.7–3.3× as long as its width, the hind femur is 5.0–5.5× as long as it is wide, and the ovipositor is 1.0–1.3× the length of the hind tibia.

Regarding the etymology, the name derives from “ruga” (Latin for “wrinkle”) and “punctus” (Latin for “puncture”), because of the largely rugose and punctate body.

#### 3.2.2. *Prochas striata* sp. nov. (Figure 4 and Figure 5)

The following features are characteristic of a female specimen. The clypeus is granulose and rugulose, while the malar space is nearly smooth. The distance between the lateral ocelli and the eyes is ca. 0.8× longer than the diameter of the ocellus. The upper half of the mesopleuron, extended toward the epicnemial carina, is striate and rugose, while the lower half is rugose and punctate, the punctures separated at most by a distance which is equal to their diameter. The scutellum and metapleuron are rugose and punctate. The scape, pedicel, mandible, and tegula are black, while the hind leg, except for the coxa, is orange brown, and the metasoma is wholly orange brown.

The following materials were examined. Holotype: CHINA • ♀; Yunnan, Xishuangbanna; 21.VI.2018; Malaise trap; No. 201804561 (BOLD ID: GBMNE65789-22). Paratypes: CHINA • 2♀; Yunnan, Maguan; 28.VI.2018; Malaise trap; No. 202019704 (BOLD ID: GBMNE65790-22), 202019705 (BOLD ID: GBMNE65791-22).

The following is a description of the female (Figure 4) holotype, with a body length of 7.4 mm and a fore wing length of 5.0 mm.

The head is characterized by an antenna with 29 flagellomeres, with the first flagellomere ca. 3.5× longer than it is wide. The face (Figure 5E) is rugose and punctate. The clypeus (Figure 5E) has a transverse carina medially, but this is abruptly declivous apically, as well as granulose, rugulose, and convex, while the apical margin is truncate and reflexed. The malar space is nearly smooth, its length ca. 0.6× the basal width of the mandible. The upper tooth of the mandible is equal to the lower tooth, with a strong lamella. The frons are rugose and punctate, without a median carina. The vertex (Figure 5F) is granulose. The interocular region is rugose and punctate. The interocular distance is 2.8× the ocello-ocular distance and 2.3× the distance between the median and lateral ocelli, while the distance between the lateral ocelli and the eyes is ca. 0.8× longer than the diameter of the ocellus. The temple mat is ca. 0.45× as long as the eyes in the lateral view. The occipital carina is evenly arched, reaching the hypostomal carina at the mandible’s base.

In the mesosoma, the pronotum is punctate dorsally, while it is transversely striate laterally. The mesoscutum (Figure 5G) is weakly rugose and punctate on a granulose surface; the notauli are absent. The scutellum and the metanotum are rugose and punctate. The mesopleuron (Figure 5B) is transversely striated below the tegula, while the upper half, extended toward the epicnemial carina, is striate and rugose, and the lower half is rugose and punctate, with punctures separated by, at most, a distance equal to their diameter. The speculum is smooth and shiny. The metapleuron is rugose and punctate. The propodeum has an area basalis that is short and trapezoid, while the superomedial area is hexagonal, ca. 0.85× as long as it is wide and separated from the area petiolaris. The superomedial area is rugose, while the area petiolaris, area dentipara, area externa, and area lateralis are rugose and punctate; all carinae are strongly developed. The propodeal spiracle is oval, connected to the pleural carina by a distinct carina.

The fore wing (Figure 5A) lacks an areolet. The portion of vein M between 2rs-m and 2m-cu is ca. 0.4× as long as 2rs-m. The marginal cell short vein RS is ca. 2.0× longer than vein 2r&RS. Vein 1cu-a is slightly distal to M&RS by 0.2× its length. The external angles of the second discal cell are acute (70°). The hind wing has a declivous CU&cu-a, but 2-CU is not connected with CU&cu-a.

In the legs, the hind femur is 4.9× longer than it is wide. The inner spur of the hind tibia is ca. 0.6× as long as the first tarsomere of the hind tarsus. The tarsal claws are pectinate.

In the metasoma, the first segment (Figure 5H) does not have a glymma and is ca. 4.0× longer than the width of the postpetiole. The petiole is ca. 3.3× longer than the postpetiole. The second tergite is ca. 0.5× as long as the first tergite and 1.5× longer than its apical width. The thyridium is oval, and its distance from the basal margin of the tergite is ca. 0.6× its length. The third tergite is as long as its apical width. The ovipositor (Figure 5D) is slightly curved upward, ca. 1.2× the apical depth of the metasoma, and ca. 0.95× the length of the hind tibia, without a notch on the upper valve. The ovipositor sheath is ca. 0.4× as long as the hind tibia.

The color of this specimen is black. The scape, pedicel, mandible, and tegula are black; the fore leg with the coxa and the basal half of the trochanter are also black, while the femur is slightly brown ventrally, and the remainder of the fore leg is orange brown. The mid leg with the coxa and the trochanter are black, while the remainder of the mid leg is orange brown. The hind leg with the coxa is black, while the remainder of the hind leg is brown. The metasoma is wholly orange brown.

Upon comparative diagnosis, this species is similar to *P. shimborii*, described by Onody and Penteado-Dias in 2015, but differs from the latter by having a face which is rugose and punctate, a first flagellomere which is 3.5× longer than it is wide, a striate and rugose area in the upper half of the mesosoma, extended toward the epicnemial carina, and a fore coxa without a ventral carina. It also has a fore wing vein 1cu-a that is slightly distal to M&RS by 0.2× of its length, an ovipositor that is slightly curved upward and 0.95× as long as the hind tibia, with a sheath 0.4× as long as the hind tibia, and scape, pedicel, mandible, and tegula that are entirely black, while the metasoma is entirely orange brown.

Regarding etymology, the name derives from “stria” (Latin for “furrow, line”), because of the striated nature and rugosity of the upper half of the mesosoma, extending upward, toward the epicnemial carina.

### 3.3. Molecular Analysis

A total of 26mt COI sequences of four *Prochas* species were used for phylogenetic analysis, of which nine sequences of two Chinese *Prochas* species were first sequenced in this study. The other 17 sequences were downloaded from the Barcode of Life Data Systems (BOLD) [35]. A COI matrix with a length of 608 base pairs was obtained based on these 26 mt COI sequences after alignment and trimming, with no insertions or deletions.

**Figure 6:**
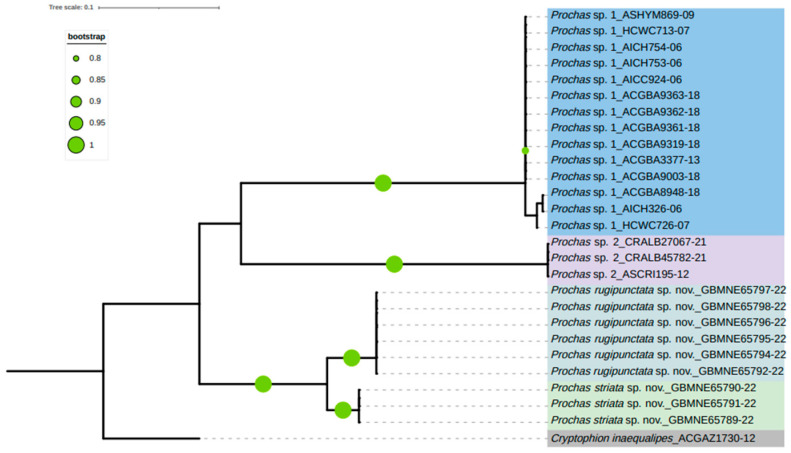
Bootstrap consensus tree generated with the maximum-likelihood (ML) method, showing the genetic relationship between isolated *Prochas* species obtained by COI sequences. Each species is highlighted with a different color. *Cryptophion inaequalipes* (ACGAZ1730-12) is used as the outgroup.

The COI analysis revealed two major clades, one of which contains all Costa Rican species, and another one which is formed of species collected in China. Chinese *Prochas* species come from different regions, but most Costa Rican specimens are from Guanacaste. *Prochas rugipunctata* sp. nov. and *Prochas striata* sp. nov. are closely related and form a sister group, and two Costa Rican species are also closely related and positioned as a sister group. The species delimitation results from ABGD and the phylogenetic analysis show two new species from China, in agreement with the morphological study (Figure 6, Dataset S1). The initial partition and recursive partition under the K2p model are shown in Figure S1.

**Figure 7:**
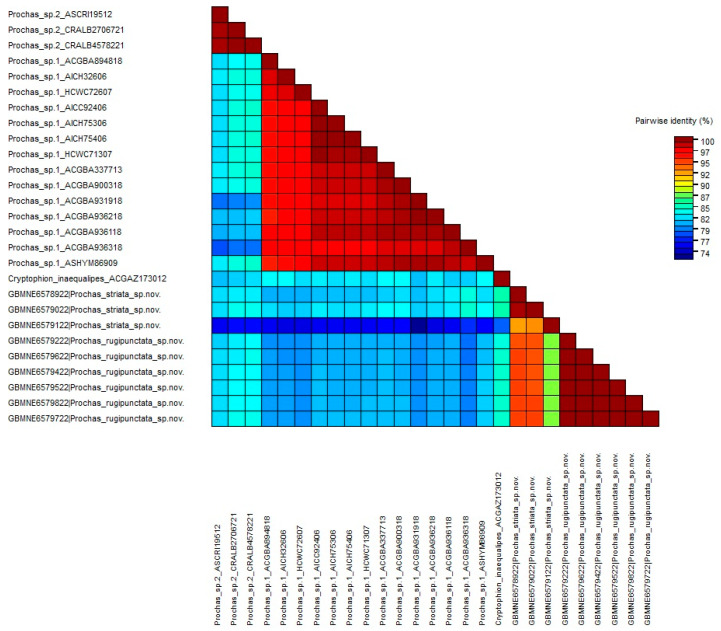
Color-coded matrix of pairwise similarity scores.

The intraspecific genetic distances varied between 0% and 4.1%, with the greatest intraspecific genetic distance of 4.1% observed in *Prochas* sp. 1. The interspecific distances ranged from 5.0% (between *Prochas striata* sp. nov. and *Prochas rugipunctata* sp. nov.) to 26.3% (between *Prochas* sp. 1 and *Prochas* sp. 2). The Kimura-2-parameter (K2P) genetic distances of interspecies and intraspecies are summarized in Table S1. The sequence consistency relationships of *Prochas* were visualized using SDTv1.3, as shown in Figure 7.

## 4. Discussion

The mitochondrial cytochrome oxidase I (*COI*) gene was hereby applied for the first time in the taxonomic analysis of the subfamily Campopleginae. Typically, the genetic divergence within species for COI gene sequences is between 0% and 2%. Our maximum intraspecific genetic distance was 4.1%, which exceeds the conventional 2% threshold. Gibson et al.’s research indicates that geographical isolation can result in genetic distances exceeding 5%, as seen in the Japanese *Eupelmus kiefferi* [36]. Our research contributes to the ongoing discourse on species delineation by demonstrating that genetic distance alone may not be a definitive criterion for species separation. A holistic approach that includes morphological characters, ecological niches, and behavioral traits is essential for a thorough assessment [37].

With only three species described before this study, our understanding of the diversity within *Prochas* is undoubtedly in its infancy. We downloaded seventeen COI sequences from the BOLD system, belonging to unidentified species, forming two distinct clades. They may represent two undescribed species from Costa Rica.

The discovery of the two new species in China resulted in a rare disjunct distribution pattern, with species known from neotropical and eastern palearctic realms, but not from intermediate areas. A biologically disjunct distribution is primarily caused by historical climate change, geographic barriers, and limited dispersal abilities [38,39]. However, a global survey of this genus would be required to reach a definitive conclusion. Broad estimates show that only a small portion of the large diversity of the subfamily Campopleginae has been described, and this subfamily may be the second largest in Ichneumonidae [20]. As such, we provisionally regard this disjunctive distribution pattern as the result of the insufficient study of this subfamily.

The phylogenetic relationships between *Prochas* and the other genera remain unclear. In Miah’s preliminary morphological analyses of Campopleginae genera, *Prochas* was recovered alongside *Cryptophion*. This result is doubtful, for there is no resolution in most groups with a low bootstrap value, and the genus *Prochas* is morphologically dissimilar to the latter [40]. Quicke et al. investigated the phylogeny of Ichneumonidae, and one undescribed species of the genus *Prochas* from Belize (northern neotropical region) was included in their analysis [41]. *Prochas* was grouped as a sister lineage to the remaining Campoplegine in this study. Currently, it is still extremely hard to discuss the systematic position of *Prochas* as little is known about the phylogeny of Campopleginae. More research is needed to clarify the relationship between *Prochas* and other genera.

## Figures and Tables

**Table 1:** The mitochondrial cytochrome c oxidase subunit (COI) sequences for molecular analysis.

BOLD ID	Species	Locality	BIN ID
GBMNE65794-22	*P. rugipunctata* sp. nov.	China: Hubei, Dabieshan	BOLD:ADC1522
GBMNE65792-22	*P. rugipunctata* sp. nov.	China: Shaanxi, Qinling Niubeiliang Natural Reserve	BOLD:ADC1522
GBMNE65798-22	*P. rugipunctata* sp. nov.	China: Shaanxi, Foping	BOLD:ADC1522
GBMNE65797-22	*P. rugipunctata* sp. nov.	China: Shaanxi, Foping	BOLD:ADC1522
GBMNE65796-22	*P. rugipunctata* sp. nov.	China: Shaanxi, Niubeiliang Guanghuojie	BOLD:ADC1522
GBMNE65795-22	*P. rugipunctata* sp. nov.	China: Zhejiang, Longquan Fengyangshan	BOLD:ADC1522
GBMNE65789-22	*P. striata* sp. nov.	China: Yunnan, Xishuangbanna	BOLD:AEY8295
GBMNE65790-22	*P. striata* sp. nov.	China: Yunnan Maguan	BOLD:AEY8295
GBMNE65791-22	*P. striata* sp. nov.	China: Yunnan Maguan	N/A
ACGBA3377-13	*Prochas* sp. 1	Costa Rica: Guanacaste, Area de Conservacion Guanacaste	BOLD:AAD2918
ACGBA8948-18	*Prochas* sp. 1	Costa Rica: Guanacaste, Area de Conservacion Guanacaste	BOLD:AAD2917
ACGBA9003-18	*Prochas* sp. 1	Costa Rica: Guanacaste, Area de Conservacion Guanacaste	BOLD:AAD2918
ACGBA9319-18	*Prochas* sp. 1	Costa Rica: Guanacaste, Area de Conservacion Guanacaste	BOLD:AAD2918
ACGBA9361-18	*Prochas* sp. 1	Costa Rica: Guanacaste, Area de Conservacion Guanacaste	BOLD:AAD2918
ACGBA9362-18	*Prochas* sp. 1	Costa Rica: Guanacaste, Area de Conservacion Guanacaste	BOLD:AAD2918
ACGBA9363-18	*Prochas* sp. 1	Costa Rica: Guanacaste, Area de Conservacion Guanacaste	BOLD:AAD2918
AICC924-06	*Prochas* sp. 1	Costa Rica: Guanacaste, Area de Conservacion Guanacaste	N/A
AICH326-06	*Prochas* sp. 1	Costa Rica: Guanacaste, Area de Conservacion Guanacaste	BOLD:AAD2917
AICH753-06	*Prochas* sp. 1	Costa Rica: Guanacaste, Area de Conservacion Guanacaste	BOLD:AAD2918
AICH754-06	*Prochas* sp. 1	Costa Rica: Guanacaste, Area de Conservacion Guanacaste	BOLD:AAD2918
ASHYM869-09	*Prochas* sp. 1	Costa Rica: Guanacaste, Area de Conservacion Guanacaste	BOLD:AAD2918
HCWC713-07	*Prochas* sp. 1	Costa Rica: Guanacaste, Area de Conservacion Guanacaste	BOLD:AAD2918
HCWC726-07	*Prochas* sp. 1	Costa Rica: Guanacaste, Area de Conservacion Guanacaste	N/A
CRALB27067-21	*Prochas* sp. 2	Costa Rica: Puntarenas, Area de Conservacion La Amistad-Pacifico	BOLD:AEO1705
CRALB45782-21	*Prochas* sp. 2	Costa Rica: Puntarenas, Area de Conservacion La Amistad-Pacifico	BOLD:AEO1705
ASCRI195-12	*Prochas* sp. 2	Costa Rica: Puntarenas, San Luis Monteverde	BOLD:AEO1705
ACGAZ1730-12	*Cryptophion inaequalipes*	Costa Rica: Guanacaste, Area de Conservacion Guanacaste	BOLD:AAR4722

## Data Availability

The COI genes newly sequenced in this study were submitted to the GenBank database under the accession numbers mentioned in Material and Methods.

## Supplementary Materials

The following supporting information can be downloaded at: https://www.mdpi.com/article/10.3390/insects15120968/s1, Figure S1: Automatic partition results by AGBD based on COI; Table S1: pairwise genetic distances COI gene suquences of *Prochas* using the K2P; Dataset S1: ABGD results based on K2P model.
