# Supplementary material for: DNA Barcodes and Morphology Reveal Two New Species of the Genus Prochas Walkley, 1959 (Ichneumonidae, Campopleginae), from China
Source: Insects. 2024 Dec 4;15(12):968. doi: 10.3390/insects15120968 (PMC11676181; doi:10.3390/insects15120968)
Supplement: Supplementary file 1 [file insects-15-00968-s001.zip › insects-3296908 Supplementary/Table S1.pdf]

COI	1	2	3	4	5	6	7	8	9	10	11	12	13	14	15	16	17	18	19	20	21	22	23	24	25	26
1																										
2	0.035																									
3	0.002	0.037																								
4	0.003	0.037	0.003																							
5	0.000	0.032	0.006	0.003																						
6	0.002	0.041	0.002	0.000	0.003																					
7	0.010	0.024	0.010	0.015	0.008	0.015																				
8	0.004	0.029	0.002	0.003	0.003	0.003	0.003																			
9	0.027	0.007	0.029	0.030	0.026	0.033	0.017	0.024																		
10	0.002	0.030	0.002	0.003	0.003	0.002	0.003	0.000	0.024																	
11	0.004	0.029	0.002	0.003	0.003	0.002	0.003	0.000	0.023	0.000																
12	0.004	0.038	0.002	0.003	0.006	0.002	0.007	0.004	0.031	0.002	0.004															
13	0.002	0.028	0.002	0.003	0.003	0.002	0.003	0.000	0.022	0.000	0.000	0.002														
14	0.016	0.004	0.018	0.018	0.014	0.020	0.010	0.013	0.004	0.013	0.013	0.020	0.011													
15	0.188	0.198	0.190	0.247	0.211	0.211	0.252	0.188	0.188	0.188	0.188	0.188	0.186	0.182												
16	0.182	0.193	0.189	0.256	0.213	0.219	0.263	0.181	0.186	0.188	0.184	0.188	0.187	0.177	0.000											
17	0.180	0.191	0.187	0.256	0.213	0.219	0.263	0.179	0.184	0.185	0.182	0.185	0.184	0.174	0.000	0.002										
18	0.193	0.207	0.196	0.207	0.195	0.200	0.208	0.194	0.206	0.197	0.197	0.194	0.195	0.192	0.196	0.193	0.193									
19	0.189	0.202	0.191	0.208	0.195	0.200	0.209	0.190	0.202	0.193	0.193	0.192	0.191	0.188	0.195	0.189	0.186	0.000								
20	0.202	0.215	0.205	0.212	0.202	0.203	0.210	0.203	0.214	0.207	0.207	0.204	0.205	0.199	0.201	0.197	0.197	0.000	0.000							
21	0.219	0.228	0.221	0.232	0.220	0.226	0.244	0.218	0.230	0.221	0.221	0.220	0.220	0.216	0.207	0.205	0.203	0.054	0.057	0.057						
22	0.217	0.225	0.219	0.232	0.217	0.223	0.244	0.215	0.228	0.218	0.218	0.218	0.217	0.214	0.204	0.203	0.200	0.052	0.055	0.055	0.002					
23	0.217	0.225	0.219	0.232	0.217	0.223	0.244	0.215	0.228	0.218	0.218	0.218	0.217	0.214	0.204	0.203	0.200	0.052	0.055	0.055	0.002	0.000				
24	0.217	0.225	0.219	0.232	0.217	0.223	0.244	0.215	0.228	0.218	0.218	0.218	0.217	0.214	0.204	0.203	0.200	0.052	0.055	0.055	0.002	0.000	0.000			
25	0.214	0.223	0.216	0.228	0.217	0.220	0.238	0.213	0.225	0.216	0.216	0.215	0.214	0.211	0.204	0.200	0.200	0.050	0.051	0.053	0.002	0.000	0.000	0.000		
26	0.214	0.223	0.216	0.228	0.217	0.220	0.238	0.213	0.225	0.216	0.216	0.215	0.214	0.211	0.204	0.200	0.200	0.050	0.051	0.053	0.002	0.000	0.000	0.000	0.000	
27	0.193	0.189	0.197	0.222	0.206	0.205	0.224	0.192	0.192	0.197	0.193	0.199	0.196	0.185	0.209	0.204	0.202	0.163	0.165	0.173	0.178	0.176	0.176	0.176	0.171	0.171

Table S1. Pairwise genetic distances COI gene sequeces of *Prochas* using the K2P. 1~14 = *Prochas* sp. 1; 15~17 = *Prochas* sp. 2; 18~20 = *Prochas striata* sp. nov.; 21~26 = *Prochas rugipunctata* sp. nov.; 27 = *Cryptophion inaequalipes*.
