# Supplementary figures and images for: DNA Barcodes and Morphology Reveal Two New Species of the Genus Prochas Walkley, 1959 (Ichneumonidae, Campopleginae), from China
Source: Insects. 2024 Dec 4;15(12):968. doi: 10.3390/insects15120968 (PMC11676181; doi:10.3390/insects15120968)

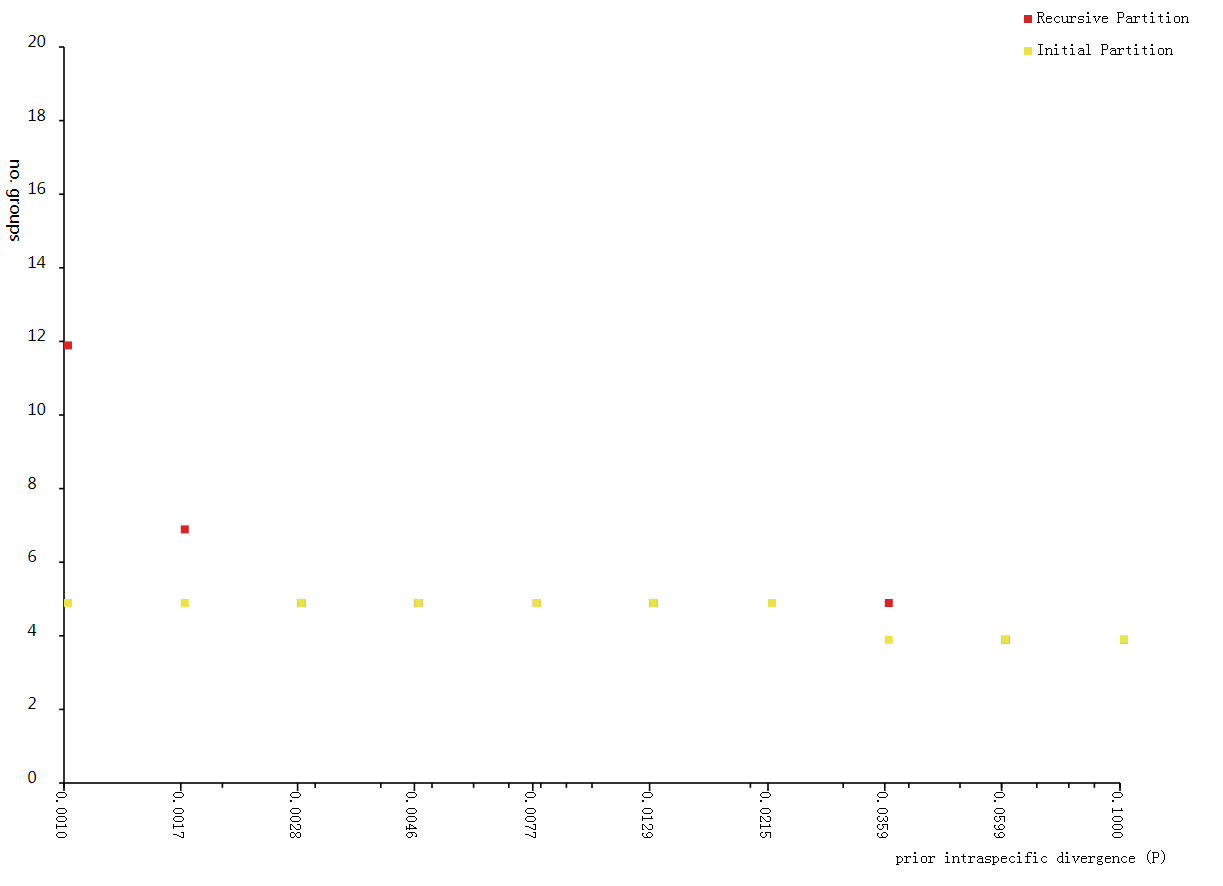

Supplement: Supplementary file 1 [file insects-15-00968-s001.zip › insects-3296908 Supplementary/Figure S1 ABGD_K2P_delimitation.png]
